# Cost-Effectiveness of Using Mass Media to Prevent Tobacco Use among Youth and Young Adults: The FinishIt Campaign

**DOI:** 10.3390/ijerph16224312

**Published:** 2019-11-06

**Authors:** Elizabeth C. Hair, David R. Holtgrave, Alexa R. Romberg, Morgane Bennett, Jessica M. Rath, Megan C. Diaz, Donna M. Vallone

**Affiliations:** 1 Schroeder Institute at Truth Initiative, Washington, DC 20001, USA; aromberg@truthinitiative.org (A.R.R.); mbennett@truthinitiative.org (M.B.); jrath@truthinitiative.org (J.M.R.); mdiaz@truthinitiative.org (M.C.D.); dvallone@truthinitiative.org (D.M.V.); 2 Department of Health, Behavior, and Society, Johns Hopkins Bloomberg School of Public Health, Baltimore, MD 21205, USA; 3 School of Public Health, University at Albany, State University of New York, Albany, NY 12144, USA; dholtgrave@albany.edu; 4 Department of Prevention and Community Health, Milken Institute School of Public Health, George Washington University, Washington, DC 20052, USA; 5 Department of Social and Behavioral Sciences, NYU College of Global Public Health, New York University, New York, NY 10012, USA

**Keywords:** tobacco control, mass media, cost effectiveness

## Abstract

Mass media campaigns have been hailed as some of the most effective tobacco prevention interventions. This study examined the cost-effectiveness of the national tobacco prevention campaign, truth^®^ FinishIt, to determine the cost per quality-adjusted life year (QALY) saved and the return on investment (ROI). The cost–utility analysis used four main parameters: program costs, number of smoking careers averted, treatment costs, and number of QALYs saved whenever a smoking career is averted. Parameters were varied to characterize cost-effectiveness under different assumptions (base case, conservative, optimistic, and most optimistic). The ROI estimate compared campaign expenditures to the cost saved due to the campaign implementation. Analyses were conducted in 2019. The base case analysis indicated the campaign results in a societal cost savings of $3.072 billion. Under the most conservative assumptions, estimates indicated the campaign was highly cost-effective at $1076 per QALY saved. The overall ROI estimate was $174 ($144 in costs to smokers, $24 in costs to the smoker’s family, and $7 in costs to society) in cost savings for every $1 spent on the campaign. In all analyses, the FinishIt campaign was found to reach or exceed the threshold levels of cost savings or cost-effectiveness, with a positive ROI. These findings point to the value of this important investment in the health of the younger generation.

## 1. Introduction

Mass media campaigns to prevent tobacco use have been identified as some of the most effective tobacco control efforts to date. These public education campaigns have contributed to the dramatic decline in tobacco use over the past few decades [1,2]. It is critical to examine the overall cost-effectiveness of these interventions, given both the significant investment required to air mass media campaigns and the potential to improve population-level health. Over the past two decades, several studies have demonstrated anti-tobacco campaigns to be cost-effective and potentially cost-saving [3,4,5,6,7]. Atusingwize et al. (2015) conducted a systematic review of tobacco control mass media campaigns from five countries, assessing their cost-effectiveness. All 10 campaigns examined were found to be cost-effective [6]. Additionally, the two federally-funded U.S. tobacco control mass media campaigns—the Food and Drug Administration’s The Real Cost, and the Centers for Disease Control and Prevention’s Tips From Former Smokers—have both been found to be cost-effective with a positive return on investment [4,5]. The extent to which mass media anti-tobacco campaigns are cost-effective or cost-saving, and how much return on investment they provide, is crucial information for policy makers, who must decide how to allocate limited tobacco control funds. Results from economic evaluations can provide benchmarks for campaign planners and help guide decisions about whether a campaign needs to change course in some way. Economic evaluations also provide frameworks for determining under what conditions a particular campaign would not be cost-saving or cost-effective [8]. Additionally, changes in tobacco use trends and media consumption among young people in recent years have resulted in changes to campaign target audiences, message content, and methods of message delivery. These changes highlight the need for economic evaluations of more recent campaigns that may differ from campaigns launched years ago in terms of their cost-effectiveness [4]. The objective of the current study was to assess the cost-effectiveness and return on investment of the recent anti-tobacco campaign, truth® FinishIt.

Truth first launched in the U.S. in 2000 with the goal of preventing smoking initiation among youth. The campaign used messages designed to expose the manipulative marketing tactics of the tobacco industry and encouraged young people to reject tobacco as a way to express their independence. Several evaluation studies found increasing exposure to the truth campaign was associated with significant strengthening of anti-tobacco attitudes, lower intentions to smoke, lower likelihood of smoking initiation, and decreases in the prevalence of youth smoking [9,10,11,12]. This campaign was also found to be cost-effective. Holtgrave et al. (2009) employed cost–utility analysis methods to assess the cost-effectiveness of the truth campaign that launched in 2000, finding that approximately $1.9 billion in medical costs were averted as a result of the campaign, far outweighing the costs associated with delivering the campaign [3].

In 2014, truth launched a new campaign called “FinishIt” aimed at accelerating the decline in youth and young adult smoking. This campaign targeted a broader audience of youth and young adults, aged 15–21, delivering messages through TV and digital platforms that were designed to increase anti-tobacco industry sentiment and reduce tobacco-related social norms and acceptability [13]. Several studies using a large, nationally representative, probability-based cohort of youth and young adults were published that indicated increasing exposure to the truth campaign is associated with stronger anti-tobacco attitudes, lower intentions to smoke, and decreased smoking prevalence among 15–21-year-olds [14,15,16]. Estimates of the effects of the FinishIt campaign as implemented indicated that the campaign was instrumental in preventing over 300,000 youth and young adults from becoming current smokers over a one-year study period [16].

A recent cost-threshold analysis of the FinishIt campaign was conducted to determine thresholds at which the campaign would be considered cost-saving or cost-effective if, in fact, the campaign cost less than the respective threshold. “Cost-saving” refers to an overall reduction is societal costs, which is directly beneficial, while “cost-effectiveness” reflects the prudent use of resources as compared to other types of interventions. The threshold analysis revealed that even with conservative assumptions regarding medical care cost savings, only 917 individuals would need to be prevented from becoming lifetime smokers in order for the campaign to be cost-effective [8]. The objective of the current study is to build on the previous cost-threshold analysis by fully examining the cost-effectiveness of the implementation of the new truth FinishIt campaign, and to examine the return on investment (ROI). This dual method approach was recently employed to examine the cost-effectiveness of the Real Cost campaign from two key vantage points [4]. Both measures allow policy makers and stakeholders to assess the value of their investment, justify campaign costs, and provide insight on what projects maximize public health outcomes. Further, by using measures of quality-adjusted life-years (QALYs) to account for both the value of additional life-years and their quality as a result of the campaign, the cost-effectiveness estimation provides further insight than the ROI.

## 2. Materials and Methods

Standard methods of cost–utility analyses were employed following the principles of the U.S. Panel on Cost-effectiveness in Health and Medicine [17], and as applied to the field of smoking prevention and cessation [3,4]. The cost–utility analysis required four main parameters: program cost (C); estimate of smoking careers averted (A); treatment costs saved whenever a smoking career is averted (T); and the number of QALYs saved whenever a smoking career is averted (Q). Using these parameters, we estimated a base case, and three other cases: conservative, optimistic, and most optimistic. The combinations of input parameter values for these cases are described below and summarized in Table 1 (which provides both input parameters and results). Decisions regarding the input parameters, described in more detail below, were based on a large body of literature examining the cost-effectiveness of health promotion interventions. Even in the base case analysis, whenever a decision had to be made on the most evidence-based parameter value from the literature, we erred on the side of caution so as to avoid biasing the results in favor of the campaign.

To fully contrast the benefits to the costs of implementing a national tobacco public education campaign, we also conducted a standard ROI analysis. We relied on the parameters and methodology used in a previous evaluation of a national tobacco public education campaign (the Real Cost campaign) [4]. This methodology required three parameters: program costs (C); estimate of smoking careers averted (A); and the total costs of smoking to society. The total costs of smoking to society was broken down by private costs (direct costs to smoker), quasi-external costs (costs to smoker’s family), and the external costs of smoking (the costs to the rest of society).

### 2.1. Cost–Utility Analysis Formulae and Parameter Values

The primary formula for the cost–utility analysis was:

Cost–utility ratio (R) = [C−AT]/[AQ].(1)

If the value of R was $0 or negative, then the campaign would be said to be cost-saving. However, a campaign may be cost-effective even if it is not cost-saving. A well-accepted standard for declaring a value of R as cost-effective is 3 × (per capita gross domestic product), or $168,348 for the U.S. [18,19]. Therefore, if R was greater than $0, but equal to or less than $168,348, the campaign would be labeled as cost-effective. If R was greater than $168,348, the campaign would not be labeled as cost-effective (although society often engages in programs which it desires but which are not cost-effective).

The value of parameter C (program costs) was described in detail in the previous cost and threshold analysis on the FinishIt campaign [8]. Briefly, the components of C include costs incurred by Truth Initiative related to the campaign development and implementation, including marketing and creative production, advertising purchases, agency fees, evaluation-related research, and Truth Initiative staff salaries. The resulting value of C was previously reported as $162,056,543 [8], and that value was used here.

The value of parameter A (smoking careers averted) was varied for more conservative and optimistic cases, based on an earlier paper examining the impact of the FinishIt campaign. This prior work estimated that 301,930 smoking careers were averted due to the campaign [16]. We used that value in the most optimistic case. However, a more conservative estimate of parameter A would take into account that not all individuals who initiate smoking go on to become lifetime smokers. Approximately 49% of those who initiate smoking between the ages of 15 and 17 years old and 46% of those who initiate between 18 and 20 years old are expected to continue to smoke after age 28 [20]. The 301,930 smoking careers were determined using data from 15–21-year-olds. Thus, a more conservative estimate of parameter A was 301,930 × 0.475 = 143,416.75. In order to introduce a high level of caution in the analysis, we used this lower value of A in the other three cases (base, conservative, and optimistic).

The value of T (treatment cost saving for each smoking career averted) has been estimated in previously published work at $22,553 [3,8,21,22]. To recap, Holtgrave et al. assumed that lifetime medical costs for a 24-year-old smoker [21] would be incurred over a 27-year period. To calculate the net present value of this cost stream he summed annual medical costs over 27 years, discounted at an annual rate of 3% and adjusted the final figure for inflation [3,8]. We employed that value for the base, optimistic, and most optimistic cases. However, some in the literature have argued that because non-smokers live longer than smokers, the lifetime medical care costs of non-smokers may be higher over their lifetime. Therefore, we used a value of $0 for T in our conservative case.

The value of Q (QALYs saved when a smoking career is averted) has been cautiously estimated in prior work examining the cost-effectiveness of tobacco prevention and cessation efforts [7,8,22,23]. We utilized Q = 1.77 for the optimistic and most optimistic cases, based on work by Villanti et al. (2012) [7] and 1.05 for the base and conservative cases, based on work by Wang et al. (2001) [24].

Generating a range of values for R across the conservative, base, optimistic, and most optimistic cases indicated whether the campaign as implemented would be considered cost-saving, cost-effective, or neither. We calculated a base case value for R, and then examined different scenarios of the impact of R by introducing both increasingly optimistic values for A, T, and Q, as well as more conservative values.

### 2.2. Return on Investment

The ROI is calculated as the benefit of the investment, divided by the cost of the investment:

ROI = [A × Individual Cost of Smoking to Society]/C.(2)

The benefit could be derived by multiplying the number of smoking careers averted (A) by the total cost of smoking to society for each smoker. The cost was equal to program costs (C). Parameters (C) and (A) were the same as those used in the base case of the cost–utility analysis.

To calculate the total costs of smoking to society for 15–21-year-olds, we used the average value for an 18-year-old smoker. We summed the private cost (cost to the smoker), quasi-external cost (cost to the smoker’s family), and external cost (cost to the rest of society) values provided in *The Price of Smoking* by Sloan and colleagues [21]. Then, we replicated the methodology used to evaluate the Real Cost campaign [4]. Non-medical values were adjusted for inflation to 2016 dollars using 1.37 for CPI less medical care costs, and medical values were adjusted using 1.77 for CPI medical care costs. Each cost was further discounted to represent the net present value of the average 18-year-old from our sample. Discounting used a value of 3% consistent with the rules and regulation of the Federal Register [25] and as used elsewhere [4]. Presented in Table 2, the total cost of smoking to society for each 18-year-old smoker is $197,071. All analyses were conducted in 2019.

## 3. Results

The estimates for R are provided in Table 1. The base case analysis indicated that the campaign resulted in a cost savings of $3.072 billion (above the cost of campaign implementation). The optimistic and most optimistic cases also showed cost savings; the most optimistic case estimated a cost-savings increase to $6.647 billion above campaign costs. In the conservative case, which omits lifetime medical costs saved due to smoking careers averted, estimates indicated that the campaign was highly cost-effective, with $1,076 per QALY saved—substantially less than the threshold of $168,348 for cost-effectiveness [18,19].

The ROI estimates are presented in Table 3. Following Equation (2) above, the investment benefit can be derived by multiplying the number of smoking careers averted (A) times the total cost of smoking to society for each smoker, approximately $28.2 billion dollars. The ROI was calculated by dividing this total benefit by the total cost of the FinishIt campaign, parameter C. For every $1 spent on the FinishIt campaign, $174 present-day dollars have been saved to society.

## 4. Discussion

In all scenarios, the effect of the FinishIt campaign on youth and young adult tobacco use was found to reach or exceed the threshold levels of either cost savings or cost-effectiveness. Moreover, most scenarios indicated that the FinishIt campaign saved billions of dollars above and beyond the cost of campaign implementation in lives saved and medical costs averted. More specifically, the return on investment calculations revealed that for every $1 spent on the campaign, $174 dollars were saved. This ROI is very similar in value to that found for the FDA Real Cost campaign ($128) [4]. As such, the overall effects of this population-based intervention highlight the value of this important investment in the health of the younger generation.

The findings are highly robust across a range of parameters and suggest the campaign implementation was well within ranges for cost-effectiveness. However, some researchers would go beyond even our conservative case and have suggested that because smokers die sooner than non-smokers, the lifetime medical costs for smokers are actually lower due to increased mortality [26]. Such a scenario posits that parameter T, the medical cost savings of a smoking career avoided should actually be negative, rather than positive or $0 as in the cases we examined. While we do not subscribe to this point of view, our findings reveal that averting a smoking career would need to incur medical costs of more than $175,000 (i.e., T = −$175,000) before the cost–utility ratio would rise above $168,000 per QALY and the campaign would no longer be considered cost-effective. Thus, the FinishIt campaign would remain cost-effective for a wide range of values across each of the parameters used in the analysis. In the same manner, if we were to only consider the external cost to smoking, not the private and quasi-external costs, our ROI estimate would still provide $7 in savings to society.

This economic evaluation, like all studies, is subject to some limitations. In particular, we must estimate rather than directly observe over time the parameters A, T, Q, and the total cost of smoking to society. These parameters are also based on prior research (utilized here for consistency across studies) and have not been updated to account for the ever-evolving changes in youth smoking patterns, medical and treatment costs, and tobacco taxes. The literature contains relatively few estimates of some parameter values (e.g., QALYs saved per smoking career averted). Wherever possible, we utilized published parameter estimates to maximize comparability with the rest of the literature (as opposed to generating new, and less comparable, parameter values). Nevertheless, the robustness of the findings to varying scenarios provides solid evidence of the cost-saving or cost-effective nature of this campaign, even assuming some uncertainty in the input parameters. Further, we are unable to quantify the unintended consequences of the campaign. For example, it is possible that adults, teachers, or parents may have changed their behavior due to being exposed to the campaign. Unfortunately, we do not have a way to quantify or monetize these changes in behavior. In addition, we have not incorporated an estimate to capture the loss in consumer surplus associated with reductions in smoking in our cost figure. Given how we adjusted the value of parameter A and that this “loss in smoking enjoyment” calculation is only relevant for established smokers, the loss in consumer surplus can be assumed to be negligible. Further, and as this debate is ongoing, we agree that consumer surplus is not a relevant parameter in economic evaluations of tobacco control interventions [27]. Lastly, our cost–utility analysis and ROI calculations treat everyone as an average, but because of health disparities across communities the FinishIt campaign may affect various populations in different manners. In other words, our estimates may underestimate the benefit to some, but it also may overestimate the benefit to others.

## 5. Conclusions

The truth campaign was established from funding through the 1999 Master Settlement Agreement (MSA), a settlement resolving the lawsuits brought by 46 U.S. states, the District of Columbia, and five territories against the major U.S. cigarette companies, to recover state Medicaid and other costs from caring for sick smokers. These findings make it clear that the resources derived from the MSA are being optimally used to prevent the disease and death brought about by tobacco use in the U.S., with a return on investment on par with other tobacco prevention campaigns for every dollar spent [4]. The truth campaign as implemented may very well be the prime example of the MSA’s success, given that the states continue to severely underfund tobacco prevention and cessation programs proven to save lives and money, despite receiving over $27 billion from the tobacco settlement and tobacco taxes [28]. Campaigns like truth can save resources for society while improving the health of generations to come.

## Figures and Tables

**Table 1:** Input parameter values and cost-utility analysis of truth® campaign. QUALY: quality-adjusted life-year.

Parameters		Base-Case Value	Source/Ref.	Optimistic Case Value	Source/Ref.	Most Optimistic Case Value	Source/Ref.	Conservative Case Value	Source/Ref.
Label	Definition								
C	Gross campaign costs	$162,056,543	[1]	$162,056,543	[1]	$162,056,543	[1]	$162,056,543	[1]
A	Number of tobacco use cases averted	143,416.75	[2,3]	143,416.75	[2,3]	301,930.00	[2,3]	143,416.75	[2,3]
T	Medical treatment costs saved per case averted	$22,553	[1]	$22,553	[1]	$22,553	[1]	--	
Q	QALYs saved per case averted	1.05	[1]	1.77	[1]	1.77	[1]	1.05	[1]
C-A × T		($3,072,421,420)		($3,072,421,420)		($6,647,370,747)		$162,056,543	
A × Q		150,588		253,848		534,416		150,588	
Cost–utility ratio (R)		($20,403)		($12,103)		($12,439)		$1076	

C = program cost, A = estimate of smoking careers averted, T = treatment costs saved whenever a smoking career is averted, Q = number of QALYs saved whenever a smoking career is averted.

**Table 2:** Cost of smoking for a smoker aged 18 years: return on investment analysis input parameters.

Cost	Private Cost (Smoker)	Quasi-External Cost (Smoker’s Family)	External Cost (Rest of Society)	Total Costs (Society as a Whole)
Cost of cigarettes	$11,588.37			$11,588.37
Federal excise taxes on tobacco	$1747.95		$(1747.95)	
State excise taxes on tobacco	$1971.76		$(1971.76)	
Mortality costs	$100,284.07			$100,284.07
Disability costs	$16,780.58			$16,780.58
Medical care cost of smoker	$1550.01		$3073.21	$4,623.22
Loss in smoker’s earnings	$25,481.32			$25,481.32
Lost income taxes on earnings			$5095.81	$5,095.81
Work loss (sick leave/absenteeism)			$3761.03	$3,761.03
Other productivity losses			$1155.74	$1,155.74
SSI ^a^ outlays and benefits	$5025.80	$(843.56)	$(4182.23)	
Private pension outlays	$6796.70	$(594.51)	$(6202.19)	
Life insurance outlays	$(8839.62)		$(8839.62)	
Spouse mortality costs (SHS ^b^)		$25,707.42		$25,707.42
Spouse disability cost (SHS)		$1199.35		$1199.35
Infant deaths (SHS)		$701.25		$701.25
Medical expenditures (SHS)		$693.21		$693.21
Totals	**$162,386.93**	**$26,863.16**	**$7821.26**	**$197,071.35**

^a^ SSI = Supplementary Security Income, ^b^ SHS = Second hand smoke.

**Table 3:** Return on investment calculation for truth^®^ campaign.

Cost Type	Costs Averted (per Smoker)	Costs Averted (all Smokers)	Return on Investment
Private costs of smoking	$162,386.93	$23,289,005,392.93	$143.71
Quasi-external costs of smoking	$26,863.16	$3,852,626,551.28	$23.77
External costs of smoking	$7821.26	$1,121,700,008.51	$6.92
Total costs of smoking	$197,071.35	$28,263,331,952.72	$174.40
